# A Novel Method of Treatment of Small Osteolabral Avulsions Associated with Posterior Fracture dislocation of the Hip

**DOI:** 10.1055/s-0042-1750714

**Published:** 2022-08-01

**Authors:** Sandeep Gupta, Rajeev Kansay, Anmol Sharma, Naveen Mittal

**Affiliations:** 1 Fortis Hospital, Mohali, Punjab, Índia; 2 Government Medical College and Hospital, Chandigarh, Índia

**Keywords:** acetabulum, femur head, fractures, bone, hip dislocation

## Abstract

Small osteolabral avulsions of the hip can be easily missed, and postreduction stress testing and a computed tomography (CT) scan of the hip should be done to look for these injuries. The usual modality of treatment of these unstable osteolabral avulsions is suture anchors, Herbert screws or spring plates. But when the bony avulsion is small, the use of these implants becomes a tedious job. We present a novel technique of fixing small osteochondral avulsion fractures not amenable to fixation using screws or spring plates.

We performed a retrospective analysis of 57 cases of patients who underwent open reduction and internal fixation for posterior fracture dislocation of the acetabulum, and we identified 6 cases of small posterior labral osteochondral fragments leading to instability. These injuries were fixed using a novelmethod. Themean Harris Hip Score at the final follow-up was of 92.5. Fixation of osteochondral avulsions associated with posterior hip fracture dislocation can be a difficult task if the bony fragment is small. Our technique is a simple, cost-effective and reliable way of fixing such avulsions with satisfactory outcomes.

## Introduction

Post-traumatic posterior hip dislocation is usually caused by high-energy trauma in young individuals involved in road traffic accidents (RTAs). The mechanism of injury is usually a dashboard injury in which there is an axially directed force from the knee in a flexed hip and, depending on the magnitude of force and position of the hip at the time of injury, several other associated injuries can occur, such as osteochondral avulsion, posterior wall fracture, and transverse fracture with posterior wall involvement. 1 Reduction of hip dislocation should be done on an emergency basis within 12 hours to decrease the risk of avascular necrosis of the hip. Nonconcentric reduction or persistent instability after reduction are usually caused by intra-articular loose bodies, an incarcerated fragment, or posterior osteolabral avulsion. 2 Addressing these injuries is of paramount importance to achieve a stable and concentric reduction to decrease the risk of subsequent redislocation of the hip, osteoarthritis, and avascular necrosis (AVN) of the hip and allow early mobilization. Small osteolabral avulsions can be easily missed, and postreduction stress testing and computed tomography (CT) scan of the hip should be done to look for these injuries. 3 The usual modality of treatment of these unstable osteolabral avulsions is suture anchors, Herbert screws, or spring plates. 4 But when the bony avuslion is small, the use of these implants becomes a tedious job. We present a novel technique of fixing small osteochondral avuslion fractures not amenable to fixation using screws or spring plates.

## Method

Our study was a retrospective analysis of 57 cases who underwent open reduction and internal fixation for posterior fracture dislocation of acetabulum by a single surgeon at a tertiary level trauma center in north India from 2012 to 2018. Approval was obtained from the ethical committee of the institution and informed written consents were obtained from all patients for inclusion in the study and publishing of data in a scientific journal without any disclosure of personal details.

Six cases with a small posterior labral osteochondral fragment leading to instability were identified and were fixed using a novel method. All 6 patients were male, with a mean age of 36.1 years old (range 21–54 years old), and the right side was involved in 5 out of 6 patients. The mechanism of injury in all six patients was RTA and all of them had a concurrent posterior dislocation at the time of injury. Three patients had a transverse acetabular fracture with associated posterior osteolabral fracture and the other three had only osteochondral fragments associated with posterior dislocation. In the latter three patients, posterior dislocation was reduced in the emergency department under sedation, but they had persistent dynamic instability. So, they were planned for surgery and dynamic instability was confirmed by stress testing in the operation theatre under C-arm in obturator view of the hip. The patients with an associated transverse fracture were planned for open reduction according to the criteria of Matta 5 and the osteochondral fragment was addressed when persistent posterior instability was noted after fixing the transverse fracture.

**Fig. 1:**
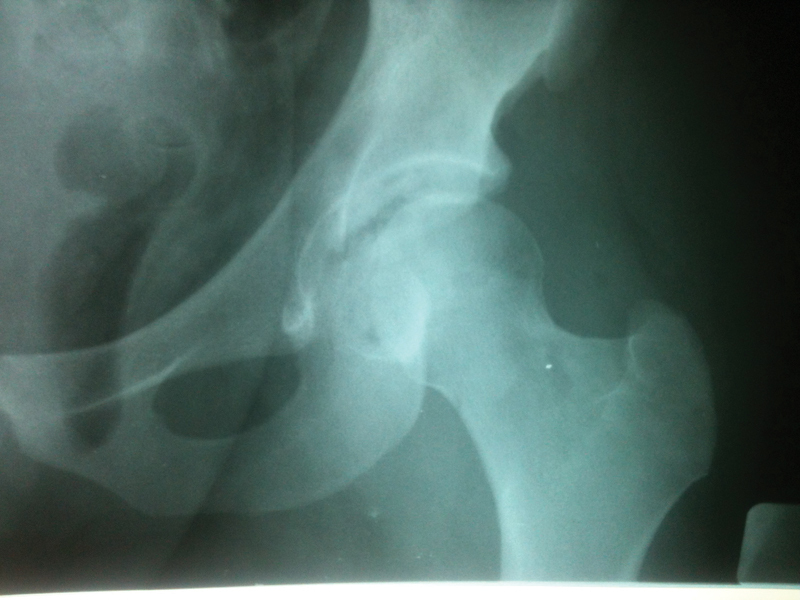
Anteroposterior view of the preoperative X ray of the left hip of a patient with small osteochondral avulsion of the posterior wall after reduction of hip dislocation.

**Fig. 2:**
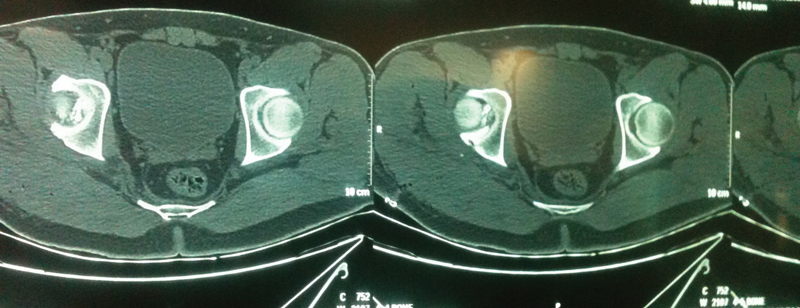
Postreduction axial images from the preoperative computed tomography scan of the affected hip.

**Fig. 3:**
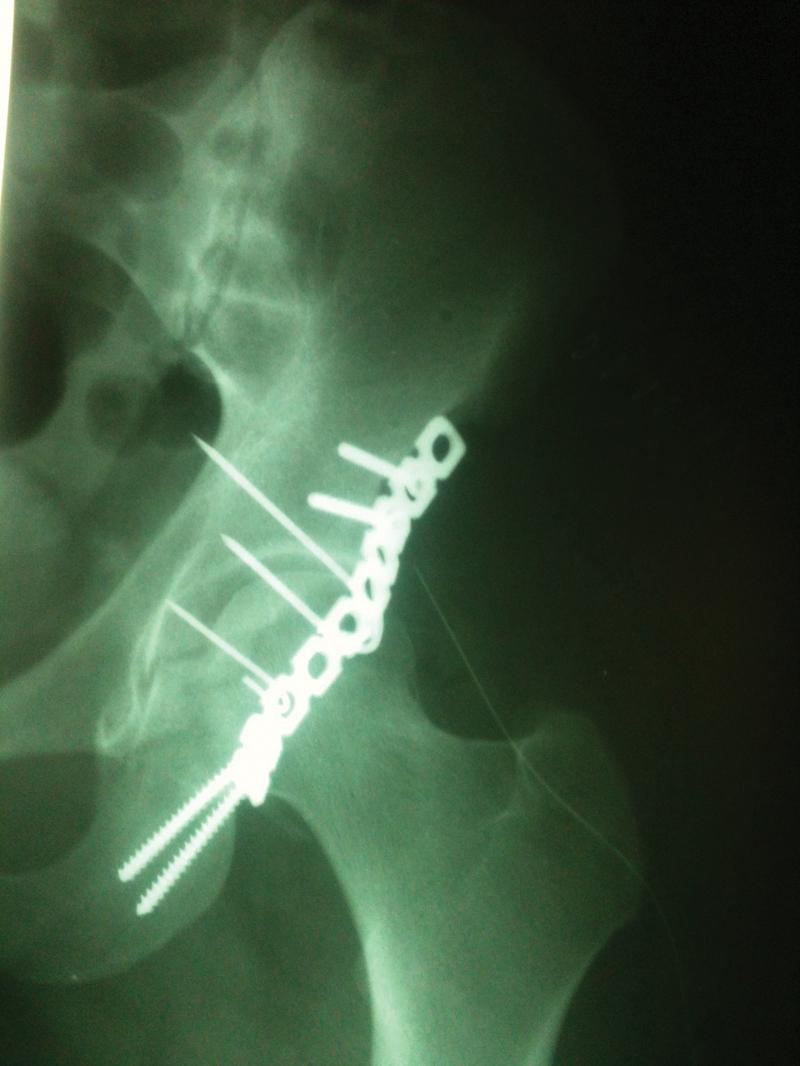
Immediate anteroposterior view of the postoperative X ray of the hip of the same patient.

All patients were operated using the Kocher Langenbeck approach. Surgical dislocation of the hip was not performed in any case. Trochanteric flip osteotomy was performed in two transverse fractures and in one posterior wall injury. The osteochondral fragments were so small in all these cases that they could not be fixed using routine methods such as screw or spring plate ( Figures 1 and 2 ). The fragments were stabilized using Kirschner wires with the lateral ends bent over the retroacetabular area and a buttress plate (3.5 mm reconstruction plate) was applied over the bent and cut hair pin loop of the Kirschner wires to secure the fixation ( Figure 3 ). Intraoperative movements were assessed immediately after fixation and the concentricity of the reduction was checked under imaging after dynamic stress testing in all views. All six hips were stable and reduced while the osteochondral fragments were also securely fixed throughout the complete range of motion of the hip.

Isometric quadriceps strengthening exercises were started immediately postoperatively. The patients were kept on toe-touch weight bearing mobilization with a walker frame for ∼ 4 weeks followed by a gradual increase in weight bearing according to the tolerance of the patient at ∼ 8 to 10 weeks after surgery, depending on the degree of radiographic consolidation of the fracture. The clinical outcome was evaluated using the Harris Hip Score at 6 weeks, 3 months, 6 months, 1 year, and at final follow-up. The quality of articular reduction and joint congruency was evaluated by postoperative plain radiographs using the Matta classification (anatomic/imperfect/poor) 5 and supplemented with 3-D CT scans. The radiological evaluation at the final follow-up was performed based on the criteria of Matta 5 : Excellent (a normal appearing hip joint); good (mild changes with minimal sclerosis and joint narrowing < 1 mm); fair (intermediate changes with moderate sclerosis and joint narrowing < 50%); and poor (advanced osteoarthritis changes). All patients were followed-up for a minimum of 2 years (mean 44 weeks; range: 24–66 weeks). There were no surgical site infections, sciatic nerve injuries, loss of reduction, or nonunion at the trochanteric osteotomy site **.** All acetabular fractures were united at the final follow-up and the mean time to union was 6.4 months (range: 4–10 months). The mean Harris Hip Score at the final follow-up was 92.5 (range: 90.4–95.8). No patient developed features suggestive of AVN hip. The radiological outcome at the final follow-up was deemed excellent in four and good in two patients.

## Final Comments

Posterior dislocation of the hip with associated wall fracture is a common injury in high energy RTAs. Persistent instability after reduction of the dislocation or fixation of the fracture should divert the attention of the orthopedist to look for any posterior osteolabral tears. Identification of a small acetabular ‘fleck sign’ in an X ray of the hip in oblique view near the posterior wall in the absence of any major acetabular fracture is a marker of posterior labral avuslion and should be assessed thoroughly by stress testing under imaging to look for any dynamic instabilities. 6 The choice of method of fixation in avulsions with small bony fragments remains arguable due to the fragment being not amenable to fixation with anchors, screws, or plates and lack of stability when fixed with Kirschner wires alone. In the present study, we described a novel technique of fixing these small osteochondral labral injuries using Kirschner wires and buttress plates over the bent wire ends. The buttress plates increased the stability of the Kirscnher wire fixation and prevented pullout of the wires. The hips remained stable after fixation on dynamic stress testing in the operation theatre under imaging and at all follow-ups. Our technique is a simple, cost-effective, and reliable way of fixing such avulsions with satisfactory outcomes.
