# Supplementary material for: A Novel Method of Treatment of Small Osteolabral Avulsions Associated with Posterior Fracture dislocation of the Hip
Source: Rev Bras Ortop (Sao Paulo). 2022 Aug 1;59(3):e467–70. doi: 10.1055/s-0042-1750714 (PMC11193590; doi:10.1055/s-0042-1750714)
Supplement: Related texts

# Research article: Um novo método de tratamento de pequenas avulsões osteolabrais associadas a fratura-luxação posterior do quadril

**Keywords:** acetábulo, cabeça do fêmur, fraturas ósseas, luxação do quadril

## Abstract

Pequenas avulsões osteolabrais do quadril podem passar despercebidas, e testes de estresse pós-redução e tomografia computadorizada do quadril devem ser realizados para sua detecção. O tratamento dessas avulsões osteolabrais instáveis geralmente é feito com âncoras de sutura, parafusos de Herbert ou placas-molas. Em avulsões ósseas pequenas, porém, o uso desses implantes é um trabalho tedioso. Apresentamos uma nova técnica de fixação de pequenas fraturas com avulsões osteocondrais não passíveis de fixação com parafusos ou placas-molas.

Realizamos uma análise retrospectiva de 57 casos de pacientes submetidos à redução aberta e fixação interna de fratura-luxação posterior do acetábulo, e identificamos 6 casos em que um pequeno fragmento osteocondral labral posterior causava instabilidade. Essas lesões foram corrigidas com um novo método. A pontuação média no Harris Hip Score no último acompanhamento foi de 92,5. A fixação de avulsões osteocondrais associadas a fratura-luxação posterior do quadril pode ser difícil se o fragmento ósseo for pequeno. Nossa técnica é uma maneira simples, econômica e confiável de corrigir tais avulsões com resultados satisfatórios.

## Introdução

A luxação posterior pós-traumática do quadril é geralmente causada por traumatismo de alta energia em indivíduos jovens que sofreram acidentes automobilísticos. Seu mecanismo é uma lesão por painel na qual há direcionamento axial de uma força do joelho para o quadril flexionado. Dependendo da magnitude da força e da posição do quadril no momento do traumatismo, várias outras lesões associadas podem ocorrer, como avulsão osteocondral, fratura da parede posterior e fratura transversal com acometimento da parede posterior. 1 A redução da luxação do quadril deve ser feita em caráter emergencial em até 12 horas para diminuir o risco de necrose avascular (NAV) do quadril. A redução não concêntrica ou instabilidade persistente após a redução é provocada por corpos soltos no espaço intra-articular, um fragmento encarcerado ou avulsão osteolabral posterior. 2 O tratamento destas lesões é de suma importância para alcançar uma redução estável e concêntrica, diminuir o risco de nova luxação subsequente, de osteoartrite e de NAV do quadril e permitir a mobilização precoce. Pequenas avulsões osteolabrais podem não ser percebidas e testes de estresse pós-redução e tomografia computadorizada (TC) do quadril devem ser realizados para sua detecção. 3 Estas avulsões osteolabrais instáveis são tratadas com âncoras de sutura, parafusos de Herbert ou placas em mola ( *spring plates* ). 4 No entanto, o uso destes implantes em pequenas avulsões ósseas é tedioso. Apresentamos uma nova técnica de fixação de fraturas com pequenas avulsões osteocondrais não passíveis de fixação com parafusos ou placas em mola.

## Método

Nosso estudo foi uma análise retrospectiva de 57 casos submetidos a redução aberta e fixação interna de fratura-luxação posterior do acetábulo por um único cirurgião em um centro de traumatismo de nível terciário no norte da Índia de 2012 a 2018. O presente estudo foi aprovado pelo comitê de ética da instituição e todos os pacientes assinaram os termos de consentimento livre e esclarecido para inclusão no estudo e publicação de dados em uma revista científica sem nenhuma divulgação de detalhes pessoais.

Identificamos seis casos nos quais um pequeno fragmento osteocondral labral posterior causava instabilidade; estes pacientes foram submetidos a fixação com o novo método. Todos os 6 pacientes eram do sexo masculino, com média de idade de 36,1 anos (variação de 21 a 54 anos); o lado direito era acometido em 5 dos 6 indivíduos. O mecanismo da lesão em todos os seis pacientes foi acidente automobilístico; todos também apresentavam luxação posterior. Três pacientes tinham uma fratura acetabular transversa com fratura osteolabral posterior e os outros três apresentavam apenas fragmentos osteocondrais associados à luxação posterior. Nestes três últimos pacientes, a luxação posterior foi reduzida no pronto-socorro sob sedação, mas houve persistência da instabilidade dinâmica. Assim, suas cirurgias foram agendadas e a instabilidade dinâmica foi confirmada por teste de esforço no centro cirúrgico com braço em C na incidência do obturador do quadril. Uma redução aberta conforme os critérios de Matta 5 foi planejada nos pacientes com fratura transversa associada; o fragmento osteocondral foi abordado quando a instabilidade posterior persistente foi observada, após a fixação da fratura transversa.

Todos os pacientes foram submetidos a cirurgia pela abordagem de Kocher Langenbeck. A luxação cirúrgica do quadril não foi realizada em nenhum caso. A osteotomia trocantérica foi realizada em duas fraturas transversas e em uma lesão de parede posterior. Em todos estes casos, o fragmento osteocondral era tão pequeno que não podia ser fixado com os métodos de rotina, como parafuso ou placa em mola ( Figuras 1 e 2 ). Os fragmentos foram estabilizados com agulhas de Kirschner com as extremidades laterais dobradas sobre a área retroacetabular e uma placa do tipo *buttress* (placa de reconstrução de 3,5 mm) foi aplicada sobre os fios dobrados e cortados como um grampo de cabelo para assegurar a fixação ( Figura 3 ). Os movimentos intraoperatórios foram avaliados imediatamente após a fixação e a concentricidade da redução foi verificada com técnicas de diagnóstico por imagem após teste de estresse dinâmico em todas as incidências. Todos os seis quadris estavam estáveis e reduzidos e o fragmento osteocondral estava firmemente fixado em toda a amplitude de movimento do quadril.

**Fig. 1:** Radiografia pré-operatória do quadril esquerdo em incidência anteroposterior de um paciente com pequena avulsão osteocondral da parede posterior após a redução da luxação do quadril.

**Fig. 2:** Imagens axiais da tomografia computadorizada pré-operatória do quadril acometido após a redução.

**Fig. 3:** Radiografia anteroposterior no pós-operatório imediato do quadril do mesmo paciente.

Exercícios isométricos de fortalecimento do quadríceps começaram imediatamente após a cirurgia. Os pacientes foram mantidos em mobilização com suporte de peso com andador por ∼ 4 semanas; posteriormente, o suporte de peso aumentou de forma gradual, de acordo com a tolerância, durante ∼ 8 a 10 semanas após a cirurgia, dependendo do grau de consolidação radiográfica da fratura. O desfecho clínico foi avaliado com o valor do Harris Hip Score em 6 semanas, 3 meses, 6 meses, 1 ano e no último acompanhamento. A qualidade da redução articular e a congruência articular foram avaliadas por radiografias simples pós-operatórias usando a classificação de Matta (anatômica/imperfeita/ruim) 5 e TCs tridimensionais. A avaliação radiológica no último acompanhamento foi realizada conforme os critérios de Matta 5 : excelente (articulação do quadril com aparência normal); boa (alterações brandas com esclerose mínima e estreitamento articular < 1 mm); regular (alterações intermediárias com esclerose moderada e estreitamento articular < 50%) e ruim (alterações avançadas de osteoartrite). Todos os pacientes foram acompanhados por um período mínimo de 2 anos (média de 44 semanas; intervalo de 24 a 66 semanas). Não houve infecção do sítio cirúrgico, lesão do nervo ciático, perda da redução ou não consolidação no sítio da osteotomia trocantérica. Todas as fraturas acetabulares estavam consolidadas no último acompanhamento. O tempo médio de consolidação foi de 6,4 meses (variação de 4 a 10 meses). A média do Harris Hip Score no último acompanhamento foi de 92,5 (intervalo de 90,4 a 95,8). Nenhum paciente desenvolveu características sugestivas de NAV do quadril. O resultado radiológico no último acompanhamento foi considerado excelente em quatro e bom em dois pacientes.

## Comentários Finais

A luxação posterior do quadril com fratura de parede é uma lesão comum em acidentes automobilísticos de alta energia. A instabilidade persistente após a redução da luxação ou da fixação da fratura deve levar à busca de qualquer laceração osteolabral posterior. A identificação de um pequeno “sinal de mancha” acetabular em radiografias oblíquas do quadril, perto da parede posterior, na ausência de qualquer fratura acetabular importante, é um marcador de avulsão labral posterior e deve levar à avaliação completa com teste de estresse em técnicas de diagnóstico por imagem para detecção de qualquer instabilidade dinâmica. 6 A escolha do método de fixação em avulsões com pequenos fragmentos ósseos é discutível, já que o fragmento não é passível de fixação com âncoras, parafusos ou placas e o uso somente de agulhas de Kirschner não oferece estabilidade. No presente estudo, descrevemos uma nova técnica de fixação destas pequenas lesões labrais osteocondrais com agulhas de Kirschner com extremidades dobradas recobertas por placas do tipo *buttress* . As placas aumentaram a estabilidade da fixação das agulhas de Kirschner e evitaram sua soltura. Após a fixação, os quadris continuaram estáveis no teste de estresse dinâmico realizado com técnicas de diagnóstico por imagem no centro cirúrgico e em todos os acompanhamentos. Nossa técnica é uma maneira simples, econômica e confiável de corrigir tais avulsões com resultados satisfatórios.
